# Children’s suggestibility for neutral arbitrary actions in the context of norm violations

**DOI:** 10.1371/journal.pone.0286241

**Published:** 2023-05-25

**Authors:** Elena Vaporova, Norbert Zmyj

**Affiliations:** Educational Sciences and Psychology, Institute of Psychology, TU Dortmund University, Dortmund, Germany; National University of Singapore Faculty of Arts & Social Sciences, SINGAPORE

## Abstract

This study investigated children’s false memories for neutral arbitrary actions. Five- to six-year-olds (*N* = 32) were taught four arbitrary actions, each following specific rules. The children then watched a televised adult performing eight actions: the four familiar actions while violating one aspect of each rule script and four unfamiliar actions. Suggestive and non-suggestive questions about all witnessed actions were asked, followed by forced-choice test questions to measure the false memory effect. The likelihood of forming false memories was higher in the suggestive condition than in the non-suggestive condition. There was no effect of previously acquired knowledge about the rules of the actions and no interaction between rule knowledge and suggestion. The results are discussed in light of previous findings in related fields of false memory research.

## Introduction

Memories do not perfectly reflect the real world. In fact, memories are prone to biases, decay and confusion, and can even be intentionally influenced by others [1]. For example, adults who have heard a fictitious story about their childhood might remember this event even though it never actually happened [2]. Memories about whole events or details of an event that were only imagined and not encoded in real life can be described as *false memories*. These false memories can occur at any age, but children are especially susceptible to suggestions by others (for a review on children’s suggestibility, see Ceci and Bruck [3]).

Research on false memories and suggestibility are relevant for fields of applied psychology such as forensic interviewing and psychotherapy, where ecological validity is crucial. Studies on false memories in adults usually employ material depicting complex scenarios that often include criminal acts (e.g., [4–8]) or otherwise emotionally charged events (e.g., [2, 9–11]). While false memory studies in children are less likely to depict criminal acts [12], they nevertheless employ complex scenarios such as a magic show [13] or a sequence from a children’s television series [14], and negatively charged events like an argument or experiencing an invasive medical procedure [9, 10, 15, 16]. For example, the Gudjonsson Suggestibility Scales [8, 17], which are a widely used test to measure interrogative suggestibility in children and adults, illustrate complex and arousing events with negative valence, such as a robbery and a couple saving a boy from an accident with his bicycle. As previous research indicates, information is processed differently depending on its valence [18], and emotions might influence false memory formation [19–21]. In one study examining the effects of emotional valence on 7- and 8-year-old children’s and adult’s memory, Kim et al. [22] found a negativity effect in children: Remembering and discrimination was better for negative than for neutral and positive words. In another study on implanting false memories in 7-year-old children, Otgaar et al. [9] found that a negative event (i.e., being accused of cheating) led to more false memories than a neutral event (i.e., moving from one classroom to another). This effect can be explained by the Paradoxical Negative Emotion Hypothesis [23] according to which negative emotion enhances both memory for true events and raises false memories.

By contrast, basic approaches in memory research use neutral arbitrary stimuli to investigate memory processes [24–27]. When investigating declarative memory from infancy to early childhood, researchers are faced with limited receptive and productive language capacities. Instead, they assess young children’s capacity to imitate arbitrary action sequences, which also targets their declarative memory (e.g., [28–32]). For example, in order to investigate long-term memory in children, Boyer et al. [30] employed an event sequence consisting of multiple actions (i.e., making spaghetti out of modeling clay). But also studies with preschoolers use neutral arbitrary stimuli to examine children’s memory [33–35]. Such studies are conducted in the laboratory using controlled designs in order to minimize possible confounding variables. While in the early days of suggestibility research, stimulus material was neutral and basic [36], a large amount of the material used in modern studies on false memory phenomena is highly complex and often arousing compared to basic memory research. In contrast, the more recent studies on suggestibility in children do not use neutral arbitrary multi-step actions as stimulus material anymore. Accordingly, our main aim was to conduct a study on false memory formation in children but to use stimuli that are common in basic research approaches when investigating memory function in children (e.g., [30, 37–39]).

Additionally, a number of studies on false memories in children have examined pre-existing knowledge about recurring events [5, 10, 15, 40–42]. This so-called script knowledge represents a generalization of what is experienced in daily life [43, 44] and is a notorious confound when recollecting episodic memory in forensic interviewing or psychotherapy. In a study investigating 6-8-year-old children’s testimony for a simulated theft, children showed a better recall for gender-role consistent characteristics exhibited by the thief than for gender-role inconsistent characteristics [4]. This selectivity in children’s reports might be explained by their use of cognitive scripts when recalling an event. Script knowledge also affects children’s false memory formation. For example, 7- and 11-year-old children’s belief that fictitious events had happened to them was found to depend on the script knowledge they had about these events: The children were more likely to form a false memory for high-script-knowledge events (i.e., finger being caught in a mousetrap) than for low-script-knowledge events (i.e., receiving a rectal enema) [10]. A later study conducted by Otgaar and colleagues [15] found that experimentally administering script knowledge about a previously unknown event also leads to an increase in false memory formation.

Given that scripts represent generalizations of what is experienced in daily life, it is difficult, by definition, to gain experimental control over these schemas. To address one aspect of script knowledge in a well-controlled experimental design, the present study focused on the role of pre-existing knowledge in false memory formation. That is, children understand the normative structure of actions and this understanding is, by definition, based on their pre-existing knowledge about how a specific action should be performed (e.g., [45, 46]). These norms can be established not only by using neutral arbitrary actions but also within a short period of time. In the seminal study of Rakoczy et al. [45], the experimenter announced that she would teach the child a game, which she labeled with an novel name. This game consisted of a series of neutral arbitrary actions involving different objects. Then, a second experimenter announced that she would perform the identical game with the same novel name. However, this second experimenter performed an action that constituted a mistake in the game. Some children spontaneously protested against this deviant action and thus demonstrated their awareness of normative structures of conventional games consisting of neutral arbitrary actions. It was evident that children not only remembered neutral arbitrary actions, but also closely monitored whether others performed the exact same actions once they were labeled with a novel name. In the present study, we were interested not only in whether children are suggestible for events that consist of neutral arbitrary actions but also whether they are suggestible when they have learned that these actions represent a norm.

We chose to test 5- to 6-year-old children since previous research indicates that younger children lack representational abilities or sufficient memory abilities, which can lead to a higher vulnerability to misleading information [14]. The children were first taught four out of a set of eight neutral arbitrary actions. Then, a three-stage misinformation paradigm (for a review, see [47]) was employed: First, the children witnessed a televised protagonist performing all eight actions while violating one critical aspect in each of the previously learned actions. Second, the children were asked questions about these actions. For half of the actions (two actions with rule knowledge, two actions without rule knowledge), suggestive questions were asked, which contained misleading information (i.e., the protagonist had allegedly performed the actions according to the previously learned rule, even though this was not the case). For the other half (two actions with rule knowledge, two actions without rule knowledge), neutral questions were asked, which did not contain misleading information. Finally, test questions about the eight actions were asked in order to measure memory performance.

In line with previous research, we expected to find a main effect of suggestion, insofar as suggestion should lead to more errors in the test questions as compared to no suggestion (hypothesis 1). Moreover, we were interested in whether previously established rule knowledge influenced the suggestibility. To the best of our knowledge, no previous studies have addressed this question using such neutral arbitrary actions. We assumed that this pre-existing knowledge about an event would make children more suggestible to misleading information that is in line with the previously established rule. Specifically, therefore, we expected an interaction effect that results in children showing a higher error rate in the suggestive condition with rule knowledge as compared to the suggestive condition without rule knowledge (hypothesis 2).

## Method

### Participants

The participants were 32 preschool children (21 girls) aged 5–6 years (age range = 5 years; 10 months– 6 years; 2 months, *M* = 5 years; 11 months; 4 days, *SD* = 23 days) from a large-sized city in Germany. We conducted a sample size calculation with an odds ratio = 3, α error probability = 0.05, Power (1 –β error probability) = 0.8 and a binomial distribution resulting in 103 necessary trials. Since each child answered four questions in each condition in a within-subject design, there were 256 trials to analyze. Additionally, ten children were tested but excluded from further analyses due to experimenter errors (*n* = 7; i.e., the experimenter used the wrong questionnaire or presented the wrong video for the respective condition), the child’s unwillingness to answer any question (*n* = 1), technical problems (*n* = 1), and the child leaving the test room during the experiment (*n* = 1). Most children (97%) were Caucasian. Sixty-nine percent of the children had one sibling, 19% had two siblings, and 13% were singletons. The parents’ educational level was as follows: 3% of the mothers had no school qualifications, 18% had a secondary school qualification, 19% had a general qualification for university entrance and 50% had a university degree; 3% of the fathers had no school qualifications, 44% had a secondary school qualification, 9% had a general qualification for university entrance and 44% had a university degree. Children received a small gift for their participation and the parents received a 5 € expense allowance. Participants were recruited from a database of parents who had agreed to be invited to participate in child development studies. Testing took place in the laboratory at the university. All parents gave their written informed consent before the experiment was conducted. The study was approved by the ethics committee of the TU Dortmund University (no. 2019–11).

### Design

The experiment had a 2 (rule knowledge: rule knowledge, no rule knowledge) x 2 (suggestion: suggestive questions, non-suggestive questions) within-subjects design. In each of the four conditions, children were asked two test questions regarding two actions, resulting in a total of eight test questions. The number of errors in these test questions was the dependent variable.

### Materials

Videos were presented on a 17-inch monitor (Iiyama ProLite, B1706S, screen resolution 1280x1024) with a DVD player (OK, OPD 200).

**Fig 1:**
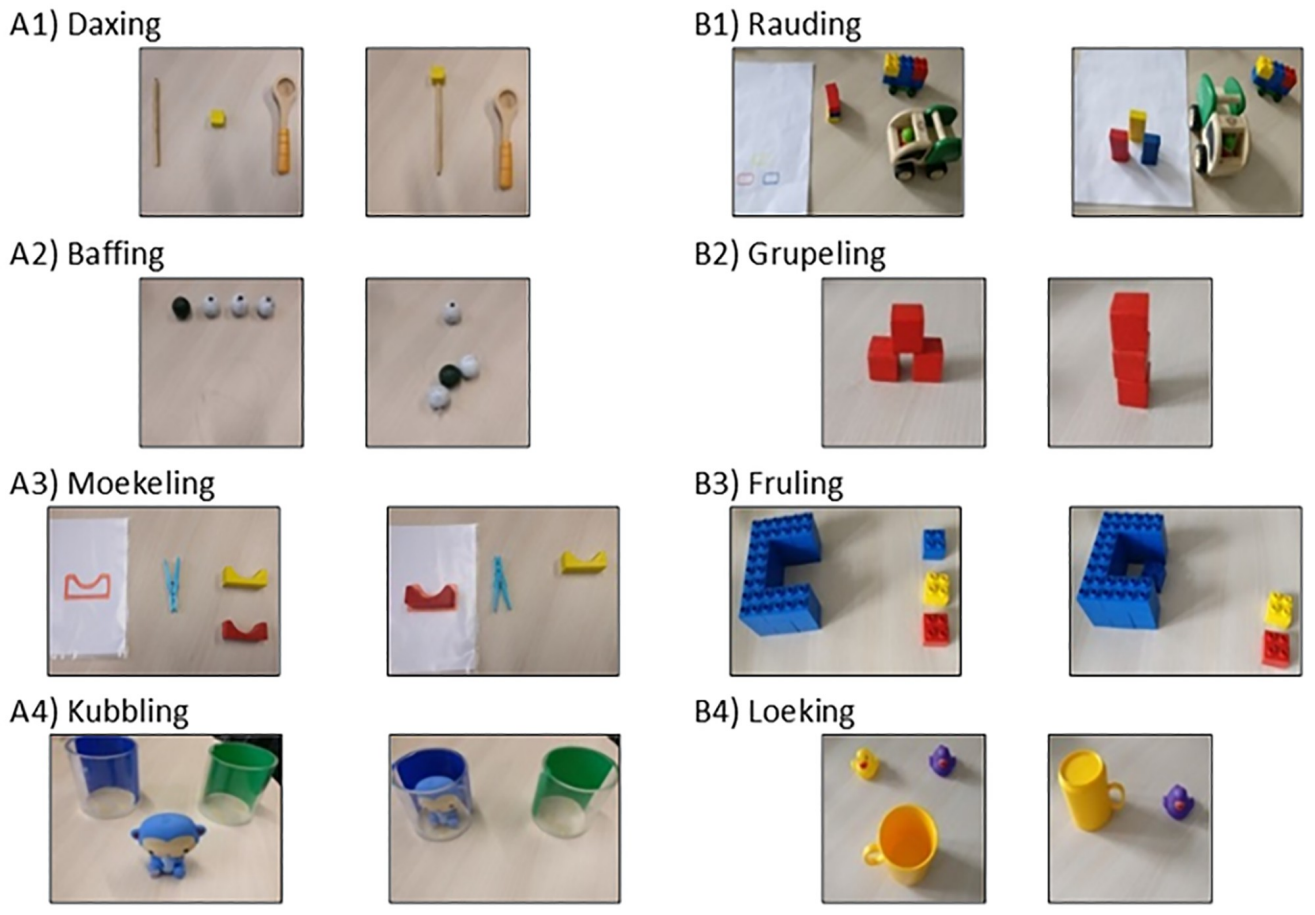
Depiction of the eight different actions used in the experiment sorted by A and B actions. *Note*. All labels are the anglicized version from the original German labels (e.g. “daxing” instead of “daxen”). A1) Daxing: Pushing the wooden cube with the pencil; script violation is pushing the wooden cube with the spoon. A2) Baffing: Stringing the wooden beads in the order white—green—white; script violation is stringing only the white beads. A3) Moekeling: Using the clothespin to lift the red wooden figure and put it on the red mark; script violation is using the clothespin to lift the yellow wooden figure and put it on the red mark. A4) Kubbling: Jumping the monkey figure on the desk twice and putting it into the blue beaker with the second jump; script violation is putting the monkey figure into the green beaker. B1) Rauding: Taking the three blocks, mounting them on the wooden dump truck, driving them to the paper and arranging them according to their color; script violation is using the Lego car instead of the wooden dump truck. B2) Grupeling: Arranging the three wooden cubes next to each other in one row, then stacking them on top of each other to build a tower; script violation is rearranging the cubes back into a pyramid. B3) Fruling: Pushing the blue brick into the blue goalpost; script violation is pushing the yellow brick into the blue goalpost. B4) Loeking: Putting the yellow mug over the yellow rubber duck; script violation is putting the yellow mug over the purple rubber duck.

All actions are depicted in Fig 1. For *daxing (we used the corresponding German translation for all eight actions*: *“[stem] + en”/+”eln”*, *e*.*g*. *“Daxen”)*, a wooden pencil (1 cm width and 17 cm height), a 4.5 cm wide and 17 cm long wooden spoon, and a 2.5 cm x 2.5 cm x 2.5 cm yellow wooden cube were used. For *baffing*, four wooden beads (diameter 3.5 cm, three white, one dark green) with a hole (diameter 1 cm) were used alongside a transparent string that was 16 cm long and had a knot at one end. For *moekeling*, a customary light blue plastic clothespin, two 7.5 cm x 2.5 cm x 2.5 cm arch-shaped wooden figures in yellow and red, and a sheet of DIN A4 paper with marks fitting the wooden figures in size and color were used. For *kubbling*, a Fisher Price Rainforest Bath Squirter figure (blue monkey) with a height of 9.5 cm and a width of 8.5 cm was used along with two round transparent plastic beakers with a diameter of 10 cm and a height of 10 cm, which had either a green or a blue film stuck on one side so that one cannot see through the beakers but can see what is inside. For *rauding*, a wooden dump truck (12.5 cm x 9 cm x 8 cm) from Wonderworld Products Co. Ltd., a self-constructed 9.5 cm x 6 cm x 5 cm car made out of Lego Duplo bricks, three 5 cm x 2.5 cm x 1 cm wooden blocks in yellow, red and blue, and a sheet of DIN A4 paper with drawn marks fitting the wooden blocks in size and color were used. For *grupeling*, 2.5 cm x 2.5 cm x 2.5 cm red wooden cubes were used. For *fruling*, a blue self-constructed 9.5 cm x 12.5 cm x 5.5 cm Lego Duplo goalpost and three 3 cm x 3 cm x 2 cm Lego Duplo bricks in yellow, blue, and red were used. For *loeking*, a yellow plastic children’s mug with a diameter of 7.5 cm and a height of 8.5 cm and two 5.5 cm x 5.5 cm x 5 cm rubber ducks in yellow and dark violet were used.

### Procedure

**Fig 2:**
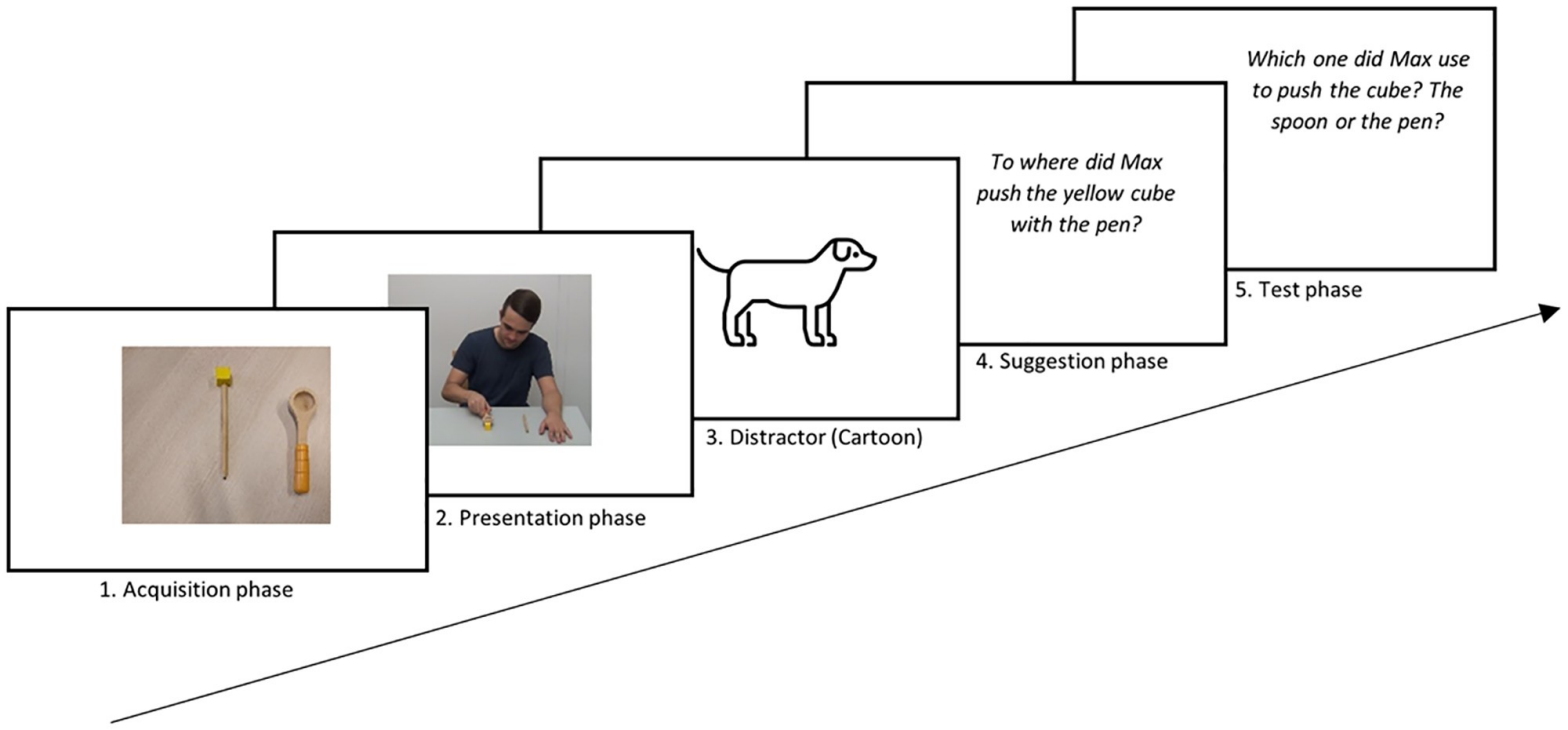
Sequence of the different phases of the procedure. *Note*. The protagonist depicted in this manuscript has given written informed consent to publish this picture.

Parent and child were welcomed by the experimenter at the entrance of the university building and guided to the test room. After a short warm-up phase, the child and the experimenter sat alone in the test room while the parent sat in an adjacent room and watched the child through a one-way mirror. The sessions were videotaped. The procedure is depicted in Fig 2.

#### Acquisition phase

The experimenter taught the child four out of eight possible actions (see “Original action in acquisitation phase” in S1 Table). Children were randomly assigned to either ‘A actions’ or ‘B actions’ (see Fig 1). For each of these actions, the experimenter first introduced the label of the action (e.g., “I will show you something. It’s called *rauding*. And *rauding* goes like this!”). Then, the experimenter demonstrated the action once (e.g., taking the three blocks, mounting them on the wooden dump truck, driving them to the paper and arranging them according to their color). Next, the experimenter demonstrated the action a second time and introduced a rule violation (e.g., using the Lego car instead of the wooden dump truck to transport the three blocks; see Fig 1) before immediately correcting herself (e.g., “Oh no, this is not how it goes! This is how it goes.”). Finally, the experimenter asked the child to imitate the action (e.g., “And now it’s your turn!”). Rule knowledge was considered as acquired when the child imitated the action correctly. Then the experimenter continued with the next action. The order of actions was fully counterbalanced across children.

#### Presentation phase

After learning the four actions (e.g., A actions), the child and the experimenter watched a protagonist performing the four actions for which the child had acquired rule knowledge in the acquisition phase (e.g., A actions) and four actions for which the child had not acquired rule knowledge (e.g., B actions, see “Alternative action shown in presentation phase” in S1 Table), in a pseudo-randomized order. The experimenter introduced the protagonist as her friend called Max, who also likes to play. The experimenter encouraged the child to watch closely because she would ask questions about the videos later. Notably, for the four acquainted actions the protagonist always performed the actions in a rule violating way (e.g., *rauding* with the Lego Duplo car instead of the wooden dump truck). After the presentation of the videos, a four-minute cartoon movie was shown as a distractor.

#### Suggestion phase

The child was then asked questions about the eight actions demonstrated by the televised protagonist. The child was told that it was okay if he/she could not remember every aspect of the actions and was encouraged to tell the experimenter if this was the case. The experimenter asked eight questions in the same order as the previously presented actions, with one question per action. There were four suggestive questions (e.g., “Can you show me to which place Max pushed the cube with the pen?”, when, in fact, he was pushing the cube with a spoon instead of a pen) and four non-suggestive questions (e.g., “Can you show me to which place Max pushed the cube?”), which were pseudo-randomized. Two suggestive and two non-suggestive questions were asked in the rule knowledge condition as well as in the no rule knowledge condition, resulting in four conditions: suggestive rule knowledge condition, suggestive no rule knowledge condition, non-suggestive rule knowledge condition and non-suggestive no rule knowledge condition.

#### Test phase

After the suggestion phase, the experimenter asked eight test questions to test for the misinformation effect. These questions were forced-choice recognition questions and were free from any suggestions (e.g., “Which object did Max use to push the cube? The pen or the spoon?”). For an overview for all questions, see S1 Table.

### Coding and data analysis

Each child answered eight test questions, resulting in a total of 256 answers. Twelve answers had to be excluded because children said that they did not know the answer. To quantify children’s false memories, they received one point for the incorrect answer and zero points for the correct answer, resulting in a maximum score of eight points in total and a maximum score of two points in each of the four conditions. The twelve excluded answers were not coded as incorrect answers. A second rater coded all of the participants. To account for this, the absolute scores were converted into percentage scores. Coding, mean error rates calculation and data preparation were conducted using IBM SPSS Statistics version 22. The logistic regression was carried out using R version 3.6.0 and RStudio version 1.2.1335. The interrater reliability between the two raters was perfect (100%).

## Results

Overall, children gave incorrect answers to 21% (*SD* = 0.41) of the test questions. In the suggestive rule knowledge condition, children gave incorrect answers in 20% (*SD* = 0.40) of all trials. In the suggestive no rule knowledge condition, children gave incorrect answers in 29% (*SD* = 0.38) of all trials. In the non-suggestive rule knowledge condition, children gave incorrect answers in 20% (*SD* = 0.40) of all trials. In the non-suggestive no rule knowledge condition, children gave incorrect answers in 17% (*SD* = 0.41) of all trials.

A two-predictor logistic model was fitted to the data to test the relationship between the likelihood of a suggestive question and an incorrect answer to the test questions (hypothesis 1), the likelihood of acquired rule knowledge and an incorrect answer to the test questions (hypothesis 2). We additionally explored the interaction between the effects of suggestive questions and acquired rule knowledge on incorrect answers to the test questions. According to the model, the log of the odds of an incorrect answer to the test questions was higher for suggestive than for non-suggestive questions (*p* < .05). The odds of an incorrect answer after being asked a suggestive question were 3.12 (= *e*^1.1375^; Table 1) times greater than after being asked a non-suggestive question. The Hosmer-Lemeshow test, conducted as a goodness-of-fit test, was not significant, indicating that the model fitted the data well, χ^2^(8) = 11.08, *p* > .05. However, the log of odds of an incorrect answer was not higher in the rule knowledge conditions than in the no rule knowledge conditions (*p* > .05, Table 1). There was no interaction effect between rule knowledge and suggestion (*p* > .05, Table 1).

**Table 1:** Logistic regression analysis of 32 children’s memory errors under the conditions rule knowledge and suggestion.

Predictor	*β*	*SE β*	Wald’s χ^2^	*p*	*e* ^*β*^
Constant	-1.82	0.39	-4.63	< .001	N.A.
Rule Knowledge	0.24	0.49	0.50	.63	1.27
Suggestion	1.14	0.46	2.47	.01	3.12
Rule Knowledge x Suggestion	-0.83	0.65	-1.29	.20	0.44

*Note*. *e*^*β*^ = odds ratio; N.A. = not applicable; *p < .05.

## Discussion

The present study was designed to investigate the suggestibility of children for neutral arbitrary actions in an experimentally highly controlled setting. Children were found to be susceptible to the experimenter’s suggestive questions, which indicates that our streamlined and controlled design is sufficient to investigate children’s suggestibility for neutral arbitrary stimuli. Previous studies in the field of developmental memory research revealed that young children are able to learn and remember neutral arbitrary multi-step action sequences observed in videos and through live performance (e.g., [45, 48, 49]). We adapted this basic approach to examine children’s suggestibility for such arbitrary and novel multi-step actions. By applying this established method of memory research in developmental psychology to false memory research, it is possible to investigate relevant factors such as familiarity with the material [50], means-end relations [51] or gist extraction [52].

The main advantage of our approach to investigate false memories is that the actions were novel for children since we used arbitrary actions in combination with novel labels. This approach is common in investigating young children’s memory [31, 37] and allows the experimental manipulation of the material. We manipulated prior expectations about what the protagonist will demonstrate, but other manipulations are possible as well. For example, one could manipulate the suggestion for different parts of actions, such as the external goal, the means to achieve this goal or the protagonist’s intention [53]. Aside from more degree of freedoms when designing experiments, another advantage of this basic approach lies in the increased control over attention capturing factors. This is important because memory and attention are interdependent: On the one hand memory is guided by attention, on the other hand attention is influenced by past experiences [54, 55]. Using short novel neutral arbitrary actions limits the influence of confounding variables in terms of attention such as emotional arousal [56, 57] or self-relevance [58, 59]. The small amount of objects against a bare neutral background makes it easier to process the presented information [60–62].

The influence of emotional valence has been discussed in various domains of memory research, with ample evidence of memory-enhancing effects of emotion, especially with respect to negative valence [9, 18, 63, 64]. This interest in the effects of emotional valence is also mirrored in false memory research [9, 65–67]. There is an ongoing debate about the extent to which emotion influences children’s false memory creation and what other developmental factors, such as working memory, might play a role [19]. One outcome of this debate is that a large proportion of false memory studies in children use emotional stimuli [4, 10, 12, 16]. The minority of studies that employed neutral stimuli applied scenarios that some children might be more familiar with than others (e.g. knowledge about a specific country, [68]; baking a cake or working on a construction site, [69]). Our goal was to introduce a paradigm in which false memories can be elicited without being emotionally arousing or familiar to the child.

In addition, we were interested in the influence of rule knowledge on false memory creation. We did not find evidence for an effect of rule knowledge, which was surprising in the light of previous findings in false memory research [4, 10, 15, 40]. This line of research shows that suggestions that tap into children’s script knowledge increase the likelihood of false memory creation. Although rule knowledge is not equivalent to script knowledge, common to both types of knowledge are expectations about the characteristics of an event. Previous studies investigating the suggestibility for false details did not manipulate script knowledge experimentally [4, 5, 40]. These studies lacked control conditions in which participants had no script knowledge about the presented events, and they did not measure pre-existing script knowledge. Furthermore, studies that reported an influence of previous knowledge on children’s false memory creation used events with a negative valence [10, 15, 42]. Information with a negative valence is preferentially processed and better integrated in memory from early childhood onwards [18], and negative events are more likely to elicit false memories compared to neutral events in children [9]. Additionally, several studies have demonstrated the impact of emotions on false memory creation [19–21]. Accordingly, the previously found effects of prior knowledge on false memory creation might be interrelated with valence or emotional arousal and do not become apparent in neutral settings.

A limitation of the current study and also possible a further explanation for the lack of influence of rule knowledge on false memory creation in the present study is that the rules established might not have entailed sufficient complexity to influence the formation of false memories in children, as is the case for scripts. A script is defined as a sequence of spatially-temporally organized expectations about actions, actors and props likely to occur during a certain event [44, 70]. The actions used in the present study were organized in a particular manner and followed certain rules, but the actions were also less complex than a real-life script such as visiting a burns center [15] or a trip to McDonald’s [40]. Script knowledge might affect the formation of false memories because the script is more complex and more elaborated, therefore leading to a higher processing fluency. A related problem might have been that not only were the rules in the present study low in complexity, but the acquisition was experienced only once. A previous study indicated that stronger memories are more resistant to suggestions than weaker memories, which the researchers manipulated through the frequency of the target presentation [69]. By contrast, another study did not find a difference in false memory formation between a script that was based on multiple experiences and a script that was based on a single experience [15]. Accordingly, future studies examining the influence of prior knowledge on false memory creation could increase the number of presentations and the complexity of a demonstrated event in order to increase the similarity to scripts.

In this study, we found a solid misinformation effect in 5- to 6-year-old children when employing neutral arbitrary stimulus material. Furthermore, we revealed that it is possible to suggest to 5- to 6-year-old children that a witnessed person followed normative structures in terms of game rules, when this was not actually the case. We found no additional effect of previously acquired rule knowledge on false memory formation. The investigation of such effects is not only relevant for a better understanding of memory processes but is also important for children’s eyewitness testimonies in legal cases, in which not only emotional memories play a role but also rather neutral memories. Future studies on false memories could use the present paradigm to experimentally manipulate aspects of an event in order to find out more about false memory formation.

## Supporting information

S1 Table List of all actions and alternative actions presented and the questions asked in the suggestion phase and the test phase. (DOCX)

## References

[1] Frenda SJ, Nichols RM, Loftus EF. Current issues and advances in misinformation research. Curr Dir Psychol Sci 2011;20:20–23.

[2] Loftus EF, Pickrell JE. The formation of false memories. Psychiatr Ann 1995;25:720–25.

[3] Ceci SJ, Bruck M. Suggestibility of the child witness: a historical review and synthesis. Psychol Bull 1993;113:403–39. 8316609 10.1037/0033-2909.113.3.403

[4] Shapiro LR, Brooks E. Effects of cognitive schemas on children’s testimony for a simulated juvenile crime. J App Dev Psychol 2018;57:1–15.

[5] Luna K, Migueles M. Typicality and misinformation: Two sources of distortion. Psicologica 2008:171–87.

[6] Shaw J, Porter S. Constructing rich false memories of committing crime. Psychol Sci 2015;26:291–301. 25589599 10.1177/0956797614562862

[7] Zaragoza MS, Mitchell KJ. Repeated exposure to suggestion and the creation of false memories. Psychol Sci 1996;7:294–300.

[8] Gudjonsson GH. A new scale of interrogative suggestibility. Pers Individ Dif 1984;5:303–14.

[9] Otgaar H, Candel I, Merckelbach H. Children’s false memories: Easier to elicit for a negative than for a neutral event. Acta Psychol 2008;128:350–54. 10.1016/j.actpsy.2008.03.009 18462700

[10] Otgaar H, Candel I, Scoboria A, Merckelbach H. Script knowledge enhances the development of children’s false memories. Acta Psychol 2010;133:57–63. 10.1016/j.actpsy.2009.09.002 19853836

[11] Toffalini E, Mirandola C, de Simone Irace C, Altoè G. False memory for pictorial scripted material: the role of distinctiveness and negative emotion. Cogn Emot 2020;34:1489–98. 32248744 10.1080/02699931.2020.1749034

[12] Lee K. Age, neuropsychological, and social cognitive measures as predictors of individual differences in susceptibility to the misinformation effect. Appl Cognit Psychol 2004;18:997–1019.

[13] Principe GF, Guiliano S, Root C. Rumor mongering and remembering: how rumors originating in children’s inferences can affect memory. J Exp Child Psychol 2008;99:135–55. 18155719 10.1016/j.jecp.2007.10.009

[14] Templeton LM, Wilcox SA. A tale of two representations: the misinformation effect and children’s developing theory of mind. Child Dev 2000;71:402–16. 10834473 10.1111/1467-8624.00153

[15] Otgaar H, Smeets T, Peters M. Children’s implanted false memories and additional script knowledge. Appl Cognit Psychol 2012;26:709–15.

[16] Jackson S, Crockenberg S. A comparison of suggestibility in 4-year-old girls in response to parental or stranger misinformation. J Appl Dev Psychol 1998;19:527–42.

[17] Gudjonsson GH. A parallel form of the Gudjonsson Suggestibility Scale. Br J Clin Psychol 1987;26:215–21. 3664038 10.1111/j.2044-8260.1987.tb01348.x

[18] Vaish A, Grossmann T, Woodward A. Not all emotions are created equal: The negativity bias in social-emotional development. Psychol Bull 2008;134:383–403. 18444702 10.1037/0033-2909.134.3.383 PMC3652533

[19] Mirandola C, Pazzaglia F. Working memory beats age: Evidence of the influence of working memory on the production of children’s emotional false memories. Front Psychol 2021;12:714498. 34484072 10.3389/fpsyg.2021.714498 PMC8416354

[20] Kaplan RL, van Damme I, Levine LJ, Loftus EF. Emotion and false memory. Emot Rev 2016;8:8–13. 10.1080/09658211.2016.1150489 26915372

[21] Porter S, Spencer L, Birt AR. Blinded by emotion? Effect of the emotionality of a scene on susceptibility to false memories. Can J Behav Sci 2003;35:165–75.

[22] Kim JM, Sidhu DM, Pexman PM. Effects of emotional valence and concreteness on children’s recognition memory. Front Psychol 2020;11:615041. 33343478 10.3389/fpsyg.2020.615041 PMC7746830

[23] Porter S, Taylor K, ten Brinke L. Memory for media: investigation of false memories for negatively and positively charged public events. Memory (Hove, England) 2008;16:658–66. 18569691 10.1080/09658210802154626

[24] Bower GH. Application of a model to paired-associate learning. Psychometrika 1961;26:255–80.

[25] von Bastian CC, Langer N, Jäncke L, Oberauer K. Effects of working memory training in young and old adults. Mem Cognit 2013;41:611–24. 10.3758/s13421-012-0280-7 23263879

[26] Engle RW, Tuholski SW, Laughlin JE, Conway ARA. Working memory, short-term memory, and general fluid intelligence: a latent-variable approach. J Exp Psychol Gen 1999;128:309–31. 10513398 10.1037//0096-3445.128.3.309

[27] Baddeley AD, Hitch G. Working Memory. In: Psychology of Learning and Motivation: Elsevier; 1974. p. 47–89.

[28] Rovee-Collier CK, Sullivan MW, Enright M, Lucas D, Fagen JW. Reactivation of infant memory. Science (New York, N.Y.) 1980;208:1159–61. 7375924 10.1126/science.7375924

[29] Gathercole SE, Pickering SJ, Ambridge B, Wearing H. The structure of working memory from 4 to 15 years of age. Dev Psychol 2004;40:177–90. 14979759 10.1037/0012-1649.40.2.177

[30] Boyer ME, Barron KL, Farrar MJ. Three-year-olds remember a novel event from 20 months: evidence for long-term memory in children? Memory (Hove, England) 1994;2:417–45. 7584302 10.1080/09658219408258957

[31] Meltzoff AN. Infant imitation after a 1-week delay: Long-term memory for novel acts and multiple stimuli. Dev Psychol 1988;24:470–76. 25147404 10.1037/0012-1649.24.4.470 PMC4137879

[32] Wang Z, Williamson RA, Meltzoff AN. Imitation as a mechanism in cognitive development: a cross-cultural investigation of 4-year-old children’s rule learning. Front Psychol 2015;6:562. 26029132 10.3389/fpsyg.2015.00562 PMC4429617

[33] Astle A, Kamawar D, Vendetti C, Podjarny G. When this means that: the role of working memory and inhibitory control in children’s understanding of representations. J Exp Child Psychol 2013;116:169–85. 23773918 10.1016/j.jecp.2013.05.003

[34] Brown AL, Scott MS. Recognition memory for pictures in preschool children. J Exp Child Psychol 1971;11:401–12. 5570449 10.1016/0022-0965(71)90045-2

[35] Fitamen C, Blaye A, Camos V. Five-year-old children’s working memory can be improved when children act on a transparent goal cue. Sci Rep 2019;9:15342. 31653944 10.1038/s41598-019-51869-4 PMC6814763

[36] Binet A. La suggestibilité. Paris: Schleicher frères; 1900.

[37] Barr R, Dowden A, Hayne H. Developmental changes in deferred imitation by 6- to 24-month-old infants. Infant Behav Dev 1996;19:159–70.

[38] Suddendorf T, Nielsen M, Gehlen R. Children’s capacity to remember a novel problem and to secure its future solution. Dev Sci 2011;14:26–33. 21159085 10.1111/j.1467-7687.2010.00950.x

[39] Krieger AAR, Aschersleben G, Sommerfeld L, Buttelmann D. A model’s natural group membership affects over-imitation in 6-year-olds. J Exp Child Psychol 2020;192:104783. 31951928 10.1016/j.jecp.2019.104783

[40] Erskine A, Markham R, Howie P. Children’s script based inferences: Implications for eyewitness testimony. Cogn Dev 2002;16:871–87.

[41] Pezdek K, Finger K, Hodge D. Planting false childhood memories: The role of event plausibility. Psychol Sci 1997;8:437–41.

[42] Pezdek K, Hodge D. Planting false childhood memories in children: The role of event plausibility. Child Dev 1999;70:887–95.

[43] Fivush R. Learning about school: The development of kindergartners’ school scripts. Child Dev 1984;55:1697. 6510051

[44] Hudson JA, Fivush R, Kuebli J. Scripts and episodes: The development of event memory. Appl Cognit Psychol 1992;6:483–505.

[45] Rakoczy H, Warneken F, Tomasello M. The sources of normativity: Young children’s awareness of the normative structure of games. Dev Psychol 2008;44:875–81. 18473651 10.1037/0012-1649.44.3.875

[46] Schmidt MFH, Rakoczy H, Tomasello M. Eighteen-month-old infants correct non-conforming actions by others. Infancy 2019;24:613–35. 32677252 10.1111/infa.12292

[47] Loftus EF. Planting misinformation in the human mind: a 30-year investigation of the malleability of memory. Learn Mem (Cold Spring Harbor, N.Y.) 2005;12:361–66. 10.1101/lm.94705 16027179

[48] McCall RB, Parke RD, Kavanaugh RD, Engstrom R, Russell J, Wycoff E. Imitation of live and televised models by children one to three years of age. Monogr Soc Res Child Dev 1977;42:1. 615290

[49] Barr R, Hayne H. Developmental changes in imitation from television during infancy. Child Dev 1999;70:1067–81. 10546335 10.1111/1467-8624.00079

[50] Kressley RA, Knopf M. A comparison of between- and within-subjects imitation designs. Inf Behav Dev 2006;29:564–73. 10.1016/j.infbeh.2006.07.012 17138309

[51] Gergely G, Bekkering H, Király I. Rational imitation in preverbal infants. Nature 2002;415:755. 10.1038/415755a 11845198

[52] Konrad C, Herbert JS, Schneider S, Seehagen S. Gist extraction and sleep in 12-month-old infants. Neurobiol Learn Mem 2016;134 Pt B:216–20. 27587286 10.1016/j.nlm.2016.08.021

[53] Tomasello M, Carpenter M, Call J, Behne T, Moll H. Understanding and sharing intentions: the origins of cultural cognition. Behav Brain Sci 2005;28:675–91; discussion 691–735. 16262930 10.1017/S0140525X05000129

[54] Chun MM, Turk-Browne NB. Interactions between attention and memory. Curr Opin Neurobiol 2007;17:177–84. 17379501 10.1016/j.conb.2007.03.005

[55] Long NM, Kuhl BA, Chun MM. Memory and Attention. In: Wixted JT, editor. Stevens’ Handbook of Experimental Psychology and Cognitive Neuroscience: Wiley; 2018. p. 1–37.

[56] Georgiou GA, Bleakley C, Hayward J, Russo R, Dutton K, Eltiti S, et al. Focusing on fear: Attentional disengagement from emotional faces. Vis Cogn 2005;12:145–58. 17460752 10.1080/13506280444000076 PMC1855164

[57] Most SB, Jungé JA. Don’t look back: Retroactive, dynamic costs and benefits of emotional capture. Vis Cogn 2008;16:262–78.

[58] Macrae CN, Visokomogilski A, Golubickis M, Sahraie A. Self-relevance enhances the benefits of attention on perception. Vis Cogn 2018;26:475–81.

[59] Cunningham SJ, Brebner JL, Quinn F, Turk DJ. The self-reference effect on memory in early childhood. Child Dev 2014;85:808–23. 23888928 10.1111/cdev.12144

[60] Broadbent D. Perception and communication. London, UK: Pergamon Press; 1958.

[61] Desimone R, Duncan J. Neural mechanisms of selective visual attention. Annu Rev Neurosci 1995;18:193–222. 7605061 10.1146/annurev.ne.18.030195.001205

[62] Lachter J, Forster KI, Ruthruff E. Forty-five years after Broadbent (1958): still no identification without attention. Psychol Rev 2004;111:880–913. 15482066 10.1037/0033-295X.111.4.880

[63] van Bergen P, Wall J, Salmon K. The good, the bad, and the neutral: The influence of emotional valence on young children’s recall. J App Res Mem Cogn 2015;4:29–35.

[64] Baltazar NC, Shutts K, Kinzler KD. Children show heightened memory for threatening social actions. J Exp Child Psychol 2012;112:102–10. 22284664 10.1016/j.jecp.2011.11.003

[65] van Damme I, Kaplan RL, Levine LJ, Loftus EF. Emotion and false memory: How goal-irrelevance can be relevant for what people remember. Memory (Hove, England) 2017;25:201–13. 26915372 10.1080/09658211.2016.1150489

[66] Melinder A, Toffalini E, Geccherle E, Cornoldi C. Positive events protect children from causal false memories for scripted events. Memory (Hove, England) 2017;25:1366–74. 28361561 10.1080/09658211.2017.1306080

[67] Bookbinder SH, Brainerd CJ. Emotion and false memory: The context-content paradox. Psychol Bull 2016;142:1315–51. 27748610 10.1037/bul0000077

[68] Candel I, Hayne H, Strange D, Prevoo E. The effect of suggestion on children’s recognition memory for seen and unseen details. Psychol Crime Law 2009;15:29–39.

[69] Pezdek K, Roe C. The Effect of Memory Trace Strength on Suggestibility. J Exp Child Psychol 1995;60:116–28.

[70] Schank RC, Abelson RP. Scripts, plans, goals and understanding: An inquiry into human knowledge structures.: Lawrence Erlbaum.; 1977.

