# Supplementary material for: Children’s suggestibility for neutral arbitrary actions in the context of norm violations
Source: PLoS One. 2023 May 25;18(5):e0286241. doi: 10.1371/journal.pone.0286241 (PMC10212140; doi:10.1371/journal.pone.0286241)
Supplement: S1 Table — (DOCX) [file pone.0286241.s001.docx]

| Action | Suggestive question | Neutral question | Test question | Original action in acquisitation phase (Fig 1) | Alternative action shown in presentation phase |
| --- | --- | --- | --- | --- | --- |
| Daxing | To where did Max push the yellow cube with the pen? | To where did Max push  the yellow cube? | Which one did Max use to  push the cube? The spoon or the pen? | Pushing the wooden cube with the pencil | Pushing the wooden cube with the spoon |
| Baffing | Where did Max put the string with the white wooden beads and the dark wooden beads after he was done? | Where did Max put the  string with the wooden beads after he was done? | What color was the wooden bead in the middle of the string? White or dark? | Stringing the wooden beads in the order white – green – white | Stringing only the white beads |
| Moekling | Which hand did Max use to  put the red wooden figure on the mark? | Which hand did Max use  to put the wooden figure on the mark? | What color was the  wooden figure that Max put on the mark with the clothespin? Red or yellow? | Using the clothespin to lift the red wooden figure and put it on the red mark | Using the clothespin to lift the yellow wooden figure and put it on the red mark |
| Kubbling | How many times did Max jump with the monkey figure before putting it into the blue beaker? | How many times did Max  jump with the monkey figure before putting it into the beaker? | What color was the beaker  into which Max jumped with the monkey figure? Blue or green? | Jumping the monkey figure on the desk twice and putting it into the blue beaker with the second jump | putting the monkey figure into the green beaker |
| Rauding | Can you show me how Max  positioned the wooden blocks from the wooden truck on the marks? | Can you show me how  Max positioned the wooden blocks on the marks? | Which vehicle did Max use  to drive the wooden blocks to the marks? The wooden truck or the Lego car? | Taking the three blocks, mounting them on the wooden dump truck, driving them to the paper and arranging them according to their color | Using the Lego car instead of the wooden dump truck |
| Grupeling | How many times did Max  reposition the three wooden blocks till he was done building a tower? | How many times did Max  reposition the three wooden blocks till he was done? | What was Max building with  the three wooden blocks? A tower, where all three blocks were on top of each other, or something different, where two blocks were on the bottom and one was on the top? | Arranging the three wooden cubes next to each other in one row, then stacking them on top of each other to build a tower | Rearranging the cubes back into a pyramid |
| Fruling | Can you show me how Max moved the blue Lego block to make the goal? | Can you show me how  Max moved the Lego block to make the goal? | What color was the Lego  block with which Max made the goal? Blue or yellow? | Pushing the blue brick into the blue goalpost | Pushing the yellow brick into the blue goalpost |
| Loeking | Do you remember on  which side Max pulled the yellow mug onto the yellow duck? | Do you remember on  which side Max pulled the yellow mug onto the duck? | What color was the duck onto  which Max pulled the mug? Yellow or violet? | Putting the yellow mug over the yellow rubber duck | Putting the yellow mug over the purple rubber duck |
